# Contribution of Ecosystem Services to Air Quality and Climate Change Mitigation Policies: The Case of Urban Forests in Barcelona, Spain

**DOI:** 10.1007/s13280-014-0507-x

**Published:** 2014-04-17

**Authors:** Francesc Baró, Lydia Chaparro, Erik Gómez-Baggethun, Johannes Langemeyer, David J. Nowak, Jaume Terradas

**Affiliations:** 1 Institute of Environmental Science and Technology (ICTA), Autonomous University of Barcelona (UAB), Campus UAB, Building C, 08193 Cerdanyola del Vallès, Barcelona Spain; 2 Ecologistas en Acción, Marqués de Leganés, 12, 28004 Madrid, Spain; 3 USDA Forest Service, SUNY-ESF, 5 Moon Library, Syracuse, NY 13210 USA; 4 Centre for Ecological Research and Forestry Applications (CREAF), Autonomous University of Barcelona (UAB), Campus UAB, Building C, 08193 Cerdanyola del Vallès, Barcelona Spain

**Keywords:** Air purification, Cities, Climate regulation, Urban ecosystem services, Urban forests, Policy targets

## Abstract

Mounting research highlights the contribution of ecosystem services provided by urban forests to quality of life in cities, yet these services are rarely explicitly considered in environmental policy targets. We quantify regulating services provided by urban forests and evaluate their contribution to comply with policy targets of air quality and climate change mitigation in the municipality of Barcelona, Spain. We apply the i-Tree Eco model to quantify in biophysical and monetary terms the ecosystem services “air purification,” “global climate regulation,” and the ecosystem disservice “air pollution” associated with biogenic emissions. Our results show that the contribution of urban forests regulating services to abate pollution is substantial in absolute terms, yet modest when compared to overall city levels of air pollution and GHG emissions. We conclude that in order to be effective, green infrastructure-based efforts to offset urban pollution at the municipal level have to be coordinated with territorial policies at broader spatial scales.

## Introduction

Urban forests, encompassing all trees, shrubs, lawns, and other vegetation in cities, provide a variety of ecosystem services to city-dwellers, such as air purification, global climate regulation, urban temperature regulation, noise reduction, runoff mitigation, and recreational opportunities, as well as ecosystem disservices, such as air quality problems, allergies, and damages on infrastructure (Escobedo et al. 2011; Gómez-Baggethun and Barton 2013; Gómez-Baggethun et al. 2013). Specifically, a significant body of literature has stressed the contribution of urban forests in reducing air pollution levels and offsetting greenhouse gas (GHG) emissions in cities (e.g., Jo and McPherson 1995; Beckett et al. 1998; McPherson et al. 1998; Nowak and Crane 2002; Yang et al. 2005; Nowak et al. 2006; Paoletti 2009; Zhao et al. 2010).

Air quality in cities is a major concern of the European Union (EU). In the last two decades, various policy instruments have been implemented at the European level to improve air quality in urban areas, mostly by regulating anthropogenic emissions of air pollutants from specific sources and sectors. These include the Directive 2010/75/EU on industrial emissions, the “Euro standards” on road vehicle emissions and the Directive 94/63/EC on volatile organic compounds emissions from petrol storage and distribution, among others. Yet, the last annual report on air quality in Europe (EEA 2013) estimated that many urban inhabitants in the EU are still exposed to air pollutant concentrations above the EU’s legally binding limits (mainly set in the Directive 2008/50/EC on ambient air quality and cleaner air for Europe). For example, the report noted that 22–33 % of the urban population within the EU was exposed to particulate matter (PM_10_) concentrations above the 24-h average limit value (50 μg m^−3^) during the period 2009–2011. This estimation of exposure increases dramatically (85–88 %) if it takes as reference the maximum levels recommended by the World Health Organization (WHO), currently set at 20 μg m^−3^ (annual mean).

As for climate change mitigation policy, the member states of the EU committed to reduce their GHG emissions by at least 20 % from 1990 levels before the end of 2020 (Climate and Energy Package, EC 2008). In an attempt to extent this commitment at the local level, the European Commission launched the “Covenant of Mayors” in 2008. This initiative involves local authorities, voluntarily committing themselves to implement more sustainable energy policies within their territories by reducing GHG emissions at the local level by at least 20 % until 2020. Such action by local authorities is deemed critical to meet global climate change mitigation targets because some 80 % of worldwide energy consumption and GHG emissions are associated with urban activities (Hoornweg et al. 2011).

The focus of urban policy-making to meet the EU targets for both air quality and climate change mitigation largely remains on technical measures such as the use of the best available technology, fuel composition requirements, energy efficiency, or renewable energy actions. The potential of urban green space in contributing to the compliance of these environmental targets is broadly neglected by urban policy-makers (Nowak 2006; Escobedo et al. 2011). Yet, a growing number of studies conclude that management of urban forests to enhance ecosystem services supply can be a cost-effective strategy to meet specific environmental standards or policy targets (e.g., Escobedo et al. 2008, 2010).

This research assesses ecosystem services and disservices provided by urban forests and it discusses their potential contribution in achieving air pollution regulation policy targets in cities. The objectives are twofold. First, we quantify in biophysical accounts and monetary values two ecosystem services (“air purification” and “global climate regulation”) and one ecosystem disservice (“air pollution” associated with biogenic volatile organic compounds (BVOC) emissions) generated by the urban forests in Barcelona, Spain. Second, we evaluate the potential of these ecosystem services to the achievement of environmental policy targets based on their actual contribution relative to air pollution and GHG emissions levels at the city scale. Accounting also the disservice allows having a “net” estimate of this contribution, since BVOC emissions from urban forests can negatively impact air quality of cities (Nowak et al. 2000).

## Materials and Methods

### Case Study: Barcelona City

**Fig. 1:**
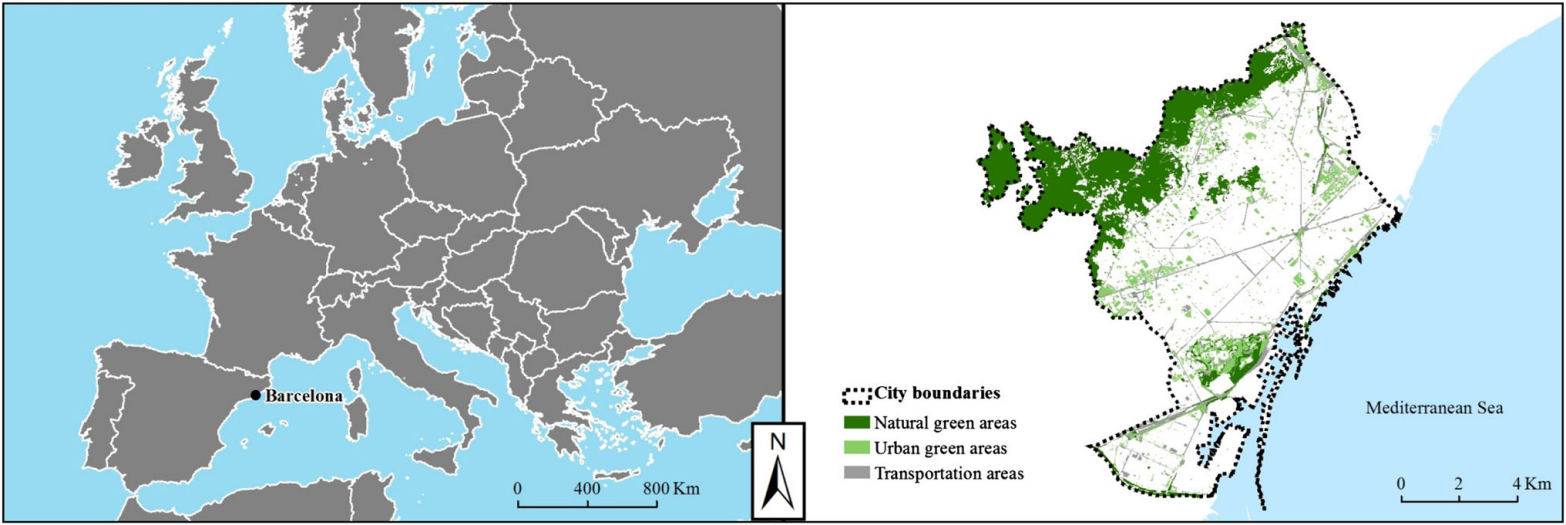
Location of Barcelona municipality and main green spaces. *Source*: Own elaboration based on Natural Earth datasets (www.naturalearthdata.com) and 3rd edition of the Ecological Map of Barcelona (Burriel et al. 2006)

We conducted our research within the administrative boundaries of the municipality of Barcelona, Spain (Fig. 1). With 1.62 million inhabitants in an area of 101.21 km^2^ (Barcelona City Council Statistical Yearbook 2012), Barcelona is the second largest city in Spain and one of the most densely populated cities in Europe (16 016 inhabitants km^−2^).

The total green space^1^ within the municipality of Barcelona amounts to 28.93 km^2^ representing 28.59 % of the municipal area and a ratio of 17.91 m^2^ per inhabitant (Barcelona City Council Statistical Yearbook 2012). Most of this green space, however, corresponds to the peri-urban forest of Collserola (protected as a natural park). The inner-city of Barcelona (excluding Collserola) embeds only 10.98 km^2^ of green space (Barcelona City Council Statistical Yearbook 2012), which amounts to 10.85 % of the municipal area and a ratio of 6.80 m^2^ of green space per inhabitant. This ratio is very low in contrast to other European cities—especially in northern countries—where green space amounts to up to 300 m^2^ per inhabitant (Fuller and Gaston 2009). Nonetheless, these low levels of green space are partly counterbalanced by the high number of single street trees, accounting for 158 896 specimens in 2011, a ratio of 98.36 street trees per 1000 inhabitants. This ratio is relatively high compared to other urban areas in Europe, which mostly ranges between 50 and 80 street trees per 1000 inhabitants (Pauleit et al. 2002). Two species, *Platanus hispanica* (46 779 trees) and *Celtis australis* (19 426 trees), account for almost one-third of the street trees in Barcelona (Barcelona City Council Statistical Yearbook 2012). Thanks to recent research (e.g., Chaparro and Terradas 2009; Terradas et al. 2011), the role of urban forests in the provision of ecosystem services in Barcelona is starting to be acknowledged by the City Council as manifested, for example, in the *Barcelona Green Infrastructure and Biodiversity Plan 2020* (2013), a planning instrument that aims to aid the development of green infrastructure^2^ (GI) strategies in the present decade.

**Fig. 2:**
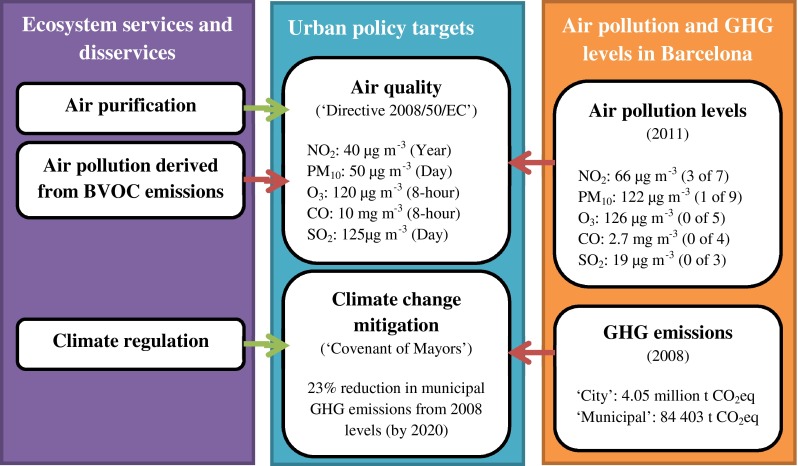
Framework for assessing links between ecosystem services and disservices, urban policy targets, and air pollution and GHG levels in Barcelona. Notes: air quality policy limits correspond to the most stringent EU values set for the protection of human health (in brackets the averaging period applicable for each limit). Some limits are subject to a specific number of allowed exceedances (e.g., PM_10_ limit can be exceeded 35 days per year at the most). See EEA (2013) for more details. Air pollution levels in Barcelona show the highest concentration values among all the monitoring stations measuring the corresponding air pollutant during the year 2011 (in brackets the number of monitoring stations exceeding the air quality limit after considering the number of allowed exceedances). See ASPB air quality report (2011) for more details. Arrows represent the links between ecosystem services and disservices, air pollution and GHG levels and urban policy targets in Barcelona (red arrows represent a negative impact towards policy targets and green arrows a positive impact). *Sources*: Own elaboration based on EEA (2013), ASPB air quality report (2011) and PECQ (2011)

As for many other large European cities (EEA 2013), air quality improvement stands as one of the major environmental policy challenges for Barcelona. In the last decade, the city has repeatedly exceeded the EU limit values for average annual concentrations of nitrogen dioxide (NO_2_) and PM_10_ pollutants (40 μg m^−3^ for both pollutants). The measures from the municipal monitoring stations during the period 2001–2011 show a steady trend for NO_2_ values and a minor decrease for PM_10_ since 2006 (ASPB air quality report 2011). During the same period, ground-level ozone (O_3_) levels have frequently exceeded the EU target value for human health (120 μg m^−3^ for a daily maximum 8-h mean period), but have never surpassed the number of allowed exceedances (25 days per year averaged over three years). Finally, carbon monoxide (CO) and sulfur dioxide (SO_2_) concentrations have been historically very low in the city of Barcelona, never exceeding the EU limit values (125 μg m^−3^ in one day for SO_2_ and 10 mg m^−3^ for 8-h average for CO) (ASPB air quality report 2011). Figure 2 synthesizes the EU limit values for air quality and the maximum levels measured in Barcelona during 2011.

In 2008, Barcelona generated approximately 4.05 million metric tons of carbon dioxide equivalent (CO_2_eq) emissions, mainly due to energy consumption in the transportation, industry, housing, and services sectors (PECQ 2011). Compared to other cities worldwide, the ratio of Barcelona (2.51 t CO_2_eq per inhabitant) is one of lowest proportions (Dodman 2009; Kennedy et al. 2009). This same year, the City Council of Barcelona signed the “Covenant of Mayors,” committing to reduce by 23 % GHG emissions only derived from services and activities directly managed by the City Council by 2020 (this so-called “municipal” GHG emissions include emissions from municipal buildings, street lighting, municipal vehicle fleet and waste collection, among others). In 2008 (baseline year for Barcelona), municipal CO_2_eq emissions amounted to 84 403 t, a ratio of 0.052 t per inhabitant (PECQ 2011, see Fig. 2).

The Energy, Climate Change and Air Quality Plan of Barcelona (PECQ 2011) provides the framework policy for air quality regulation and climate change mitigation during the period 2011–2020. Like other policy instruments aimed at improving indicators of environmental quality, the PECQ does not consider the enhancement of green infrastructure as a potential strategy to meet the policy targets established for air pollution concentrations and GHG emissions, as it focuses mainly on measures to improve energy efficiency and other technical fixes.

### Sample Design and Data Collection

The i-Tree Eco model (formerly known as Urban Forests Effects—UFORE) (Nowak and Crane 2000) was used to quantify ecosystem services and disservices in Barcelona. The i-Tree Eco model has been used in more than 50 cities across the world, especially in the United States, to assess urban forest structure and ecosystem services (Nowak et al. 2008a).

**Fig. 3:**
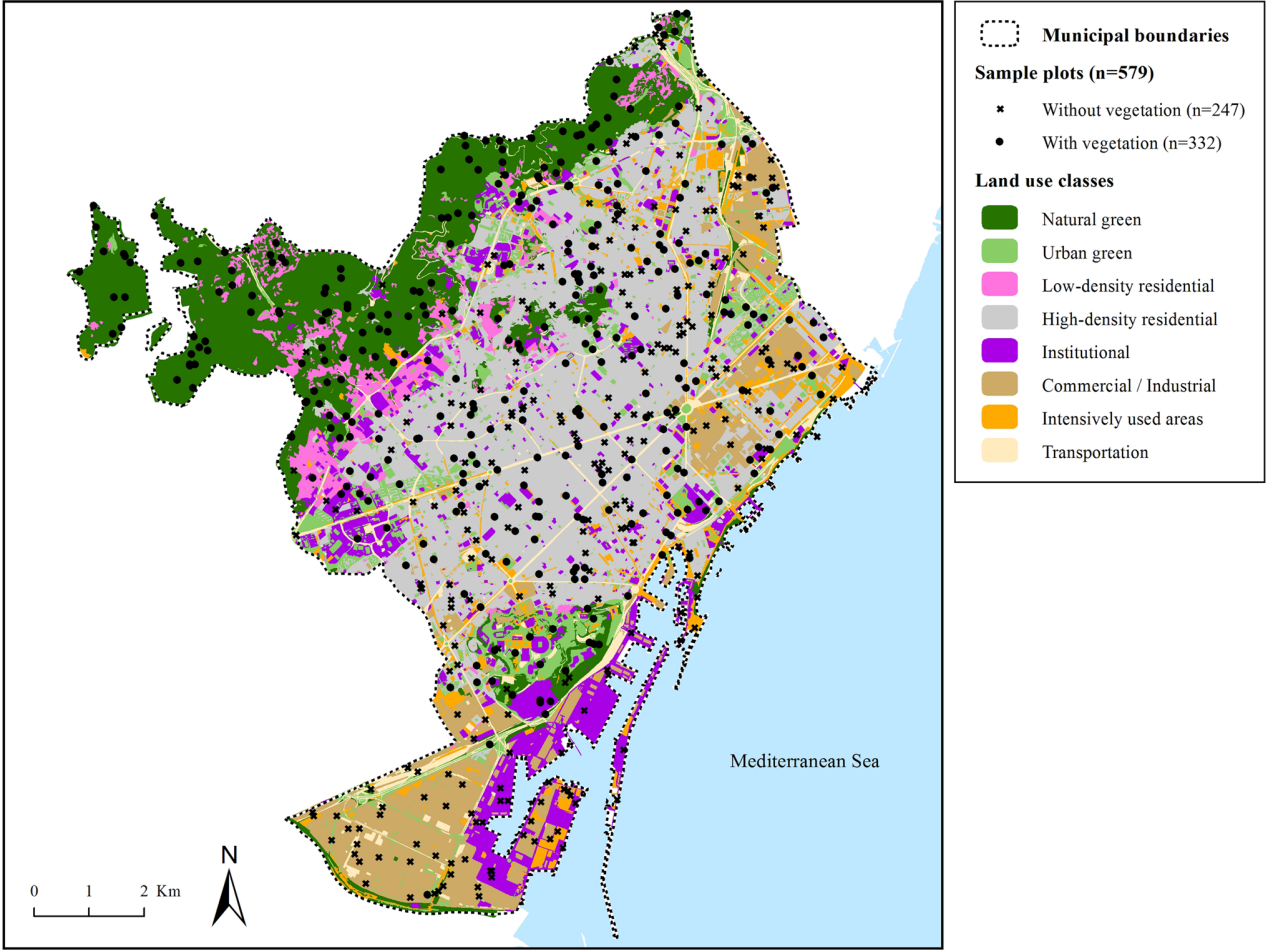
Land use classes and location of sample plots within the municipality of Barcelona. *Source*: Own elaboration based on the 3rd edition of the Ecological Map of Barcelona (Burriel et al. 2006)

I-Tree Eco protocols (Nowak and Crane 2000; Nowak et al. 2008a, b; i-Tree User’s Manual 2008) were followed to collect field data on urban forest structure within the municipality of Barcelona. Field data were collected within 579 randomly located circular plots (each measuring 404 m^2^; 11.34 m radius) distributed across the city and pre-stratified among eight land use classes based on the 3rd edition of the Ecological Map of Barcelona (Burriel et al. 2006, see Fig. 3). Plot centers were positioned from a random number generator of *x* and *y* coordinates for each land use class by means of a geographic information system (Miramon software, see Pons 2006). Prior to fieldwork, plots without vegetation cover were identified using 1:5000 digital aerial ortho-photographs from the Catalan Cartographic Institute (year 2004). Only the plots with vegetation cover (trees, shrubs or herbaceous flora) were then visited for field data collection (see Table 1 for sample data general figures).

**Table 1:** Sample data by land use stratification

Land use class	Description^a^	Total area (ha)	Sample data				
			Sampled area (ha)	No. of plots	No. of plots with woody vegetation^b^	No. of trees	No. of shrub masses^c^
Urban green	Urban parks, lawns, allotment gardens, permanent crops, flowerbeds	806	2.02	50	50	544	89
Natural green	Woodland, scrubland, grassland, riparian vegetation, bare rock	2184	5.05	125	117	1844	329
Low-density residential	1–2 family dwellings (normally with private garden)	424	0.81	20	15	174	55
High-density residential	Multi-family dwellings with or without commercial areas	3666	8.24	204	102	531	79
Transportation	Parking lots, roads, rails and streets, stations	513	1.21	30	14	69	10
Institutional	Education, health, military, sport and other public facilities, cemeteries, port	776	1.58	39	3	21	0
Commercial/industrial	Factories and other industrial areas, warehouses, large shopping centers	1185	2.83	70	7	14	0
Intensively used areas	Pedestrian areas, vacant areas, areas in transformation	567	1.66	41	24	148	8
Total		10 121	23.39	579	332	3345	570

^a^Based on land use subclasses from the 3rd edition of the Ecological Map of Barcelona (Burriel et al. 2006)

^b^Plots with woody vegetation account for those whether with shrubs or trees, or both

^c^Data on shrubs were collected for shrub masses (same species and height) and not at the individual level

Fieldwork was carried out from May to July 2009. Plots were located using a GPS device supported by high resolution maps containing the precise position of the plot center and its perimeter. Inaccessible plots (due to the steep slope, lack of permission to enter private areas, impenetrable vegetation, among others) were relocated in the closest accessible area with similar land use and vegetation characteristics. The general information collected from each visited plot included, among other parameters, date of visit, GPS coordinates, actual land use (and percent of land uses if the plot fell in more than one land use class), and percents of tree cover, shrub cover, plantable space, and ground cover. Main data on shrubs included the identification of species (genus at a minimum), average height, and percent area relative to total ground area. These data were collected for shrub masses (same species and height) and not at the individual level. Main data on trees included the identification of species, diameter at breast height (DBH), total height, height to crown base, crown width, percent of canopy missing (relative to crown volume), percent of impervious soil beneath canopy, percent of shrub cover beneath the canopy, and light exposure of the crown (see Nowak et al. 2008a for a complete list of data measures). Requirements of data inputs also include hourly air pollution concentrations and meteorological data (e.g., air temperature, solar radiation, and precipitation averages) for a complete year. The Public Health Agency of Barcelona (ASPB) provided concentration data for CO, SO_2_, O_3_, NO_2_, and PM_10_ air pollutants from the 13 operational monitoring stations of the city during the year 2008. Meteorological data of Barcelona was directly retrieved from the US National Climatic Data Centre (year 2008). Thus, the results from the evaluation of ecosystem services and disservices correspond to the year 2008.

### Quantification and Valuation of Ecosystem Services and Disservices

Field data of urban forest structure, air pollution, and meteorological data were processed using i-Tree Eco software (www.itreetools.org) to quantify the ecosystem services of air purification and climate regulation, and the disservice air pollution derived from BVOC emissions in both biophysical and economic terms. Besides, the model also provided general results on the urban forest structure of Barcelona, including information on species composition, species origin and diversity, leaf area index (LAI), and leaf biomass. The analysis of the urban forest structure of Barcelona is beyond the scope of this paper; however, we refer to some relevant information in “Discussion” section.

The air purification service was quantified on the basis of field data, air pollution concentration, and meteorological data. Fundamentally, the i-Tree Eco model estimates dry deposition of air pollutants (i.e., pollution removal during non-precipitation periods), which takes place in urban trees and shrub masses. The (removed) pollutant flux (*F*; in g m^−2^ s^−1^) is calculated as the product of deposition velocity (*V*_d_; in m s^−1^) and the pollutant concentration (*C*; in g m^−3^). Deposition velocity is a factor computed from various resistance components (for more details see Baldocchi et al. 1987; Nowak and Crane 2000; Nowak et al. 2006, 2008a). Monetary values of the ecosystem service air purification were estimated in i-Tree Eco from the median externality values for each pollutant established for the United States (Murray et al. 1994) and adjusted by the producer’s price index for the year 2007 (U.S. Department of Labor). Externality values applied to the case study are: NO_2_ = 9906 USD t^−1^, PM_10_ = 6614 USD t^−1^, SO_2_ = 2425 USD t^−1^, and CO = 1407 USD t^−1^. Externality values for O_3_ are set to equal the value for NO_2_.

The ecosystem service of climate regulation was calculated based on the modeling results of gross carbon sequestration, net carbon sequestration (i.e., estimated net carbon effect after accounting for decomposition emission of carbon from dead trees), and carbon storage. The i-Tree Eco model calculates the biomass for each measured tree using allometric equations from the literature. Biomass estimates are combined with base growth rates, based on length of growing season, tree condition, and tree competition, to derive annual biophysical accounts for carbon storage and carbon sequestration. Several assumptions and adjustments are considered in the modeling process (for more details, see Nowak and Crane 2000, 2002; Nowak et al. 2008a). To estimate the monetary value associated with urban tree carbon storage and sequestration, biophysical accounts were multiplied by 78.5 USD t^−1^carbon based on the estimated social costs of carbon dioxide emissions in the US for the year 2010 (discount rate 3 %, EPA 2010). Additionally, we considered GHG emissions generated by the municipal vehicle fleet dedicated to green space management (862.50 t CO_2_eq according to PECQ 2011) as a proxy of total GHG emissions directly attributable to green space maintenance. Hence, this measure was subtracted from total net carbon sequestration estimate provided by urban forests (after applying the conversion factor 1 g C = 3.67 g CO_2_eq).

The emission of BVOCs from trees and other vegetation can contribute to the formation of ground-level O_3_ and CO air pollutants (Kesselmeier and Staudt 1999), hence counteracting the air purification that vegetation delivers. BVOC emissions depend on factors such as tree species, leaf biomass, daylight, and air temperature (Nowak et al. 2008a). The i-Tree Eco model estimates the hourly emission of isoprene (C_5_H_8_), monoterpenes (C_10_ terpenoids), and other BVOCs by trees and shrubs species using protocols of the Biogenic Emissions Inventory System (BEIS; see Nowak et al. 2008a for further details). To estimate the amount of O_3_ produced by BVOC emissions, the model applies incremental reactivity scales (g O_3_ produced per g BVOC emitted) based on Carter (1994). CO formation from BVOC emissions is estimated for an average conversion factor of 10 % based on empirical evidence (Nowak et al. 2002a). However, due to the high degree of uncertainty in the approaches of estimating O_3_ and CO formation derived from BVOC emissions, no estimates of the total amount of pollution formed by urban forests are given (neither monetary costs). Only index values can be calculated to compare the relative impact of the different species on O_3_ and CO formation (Nowak et al. 2002a).

### Contribution of Urban Forests to Air Quality Improvement and Climate Change Mitigation

The relative contribution of urban forests to air quality improvement and climate change mitigation in Barcelona for the year 2008 was determined based on data of air pollution levels and GHG emissions. We considered emissions generated within the municipal area (hereafter city-based pollution) and pollution not directly attributable to city-based emissions (hereafter background pollution) to determine air pollution levels in the city. We only accounted for PM_10_ and NO_2_ levels since, as described above, these are the two air pollutants whose concentrations are frequently exceeding EU value limits in the city. Data for city-based pollution and background pollution were extracted from PECQ (2011) estimations. PECQ (2011) measures include aggregated and disaggregated city-based emissions from different sectors (road transport, residential and tertiary, industry and energy generation, and port activity), which in turn draws on a wide range of primary data sources (e.g., vehicle population, annual vehicle mileage, consumption of gas in households and businesses, etc.) and apply various quantitative methods (e.g., COPERT/CORINAIR model for road transport). Background pollution is measured from real pollutant concentration values recorded by the monitoring stations in the city and from one monitoring station located in the area of “Cap de Creus” (130 km north-east from Barcelona), hence not influenced by polluting activities within the city. According to PECQ (2011), the annual average concentration of NO_2_ for the year 2008 in Barcelona was mainly determined by emissions from road traffic (65.6 %), while background pollution only accounted for 18.7 %. In contrast, the annual average of the PM_10_ concentration was primarily determined by background pollution (88.1 %).

The rate of GHG emissions was also extracted from PECQ (2011). Calculations are based on the various energy sources generating GHG emissions in the city (mainly electricity, natural gas and vehicle fuels). Electricity-related GHG emissions are calculated based on the Catalan electricity mix.

## Results

### Air Purification

**Fig. 4:**
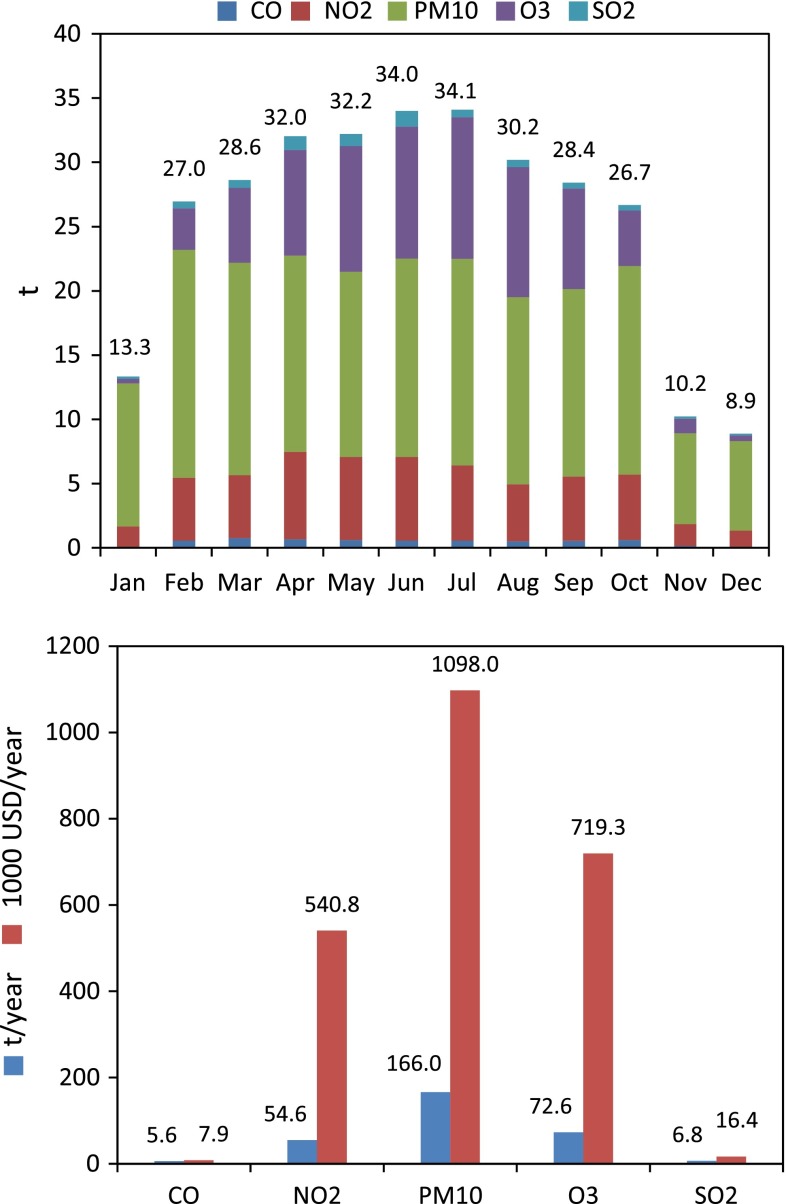
Monthly and annual air pollution removal by air pollutant (urban forests of the municipality of Barcelona, year 2008)

Total air purification is estimated at 305.6 t of removed pollutants year^−1^ with an economic value of 2.38 million USD year^−1^ (Fig. 4). PM_10_ removal is the highest among the five air pollutants analyzed (i.e., CO, NO_2_, PM_10_, O_3_, and SO_2_), accounting for 54 % of the total biophysical value (166.0 t year^−1^) and 46 % of the total economic value (1.10 million USD year^−1^). Pollution removal was lower for NO_2_ and ground-level O_3_ (54.6 t, 541 000 USD for NO_2_; 72.6 t, 719 000 USD for O_3_), and lowest for CO and SO_2_ (5.6 t, 7880 USD for CO; 6.8 t, 16 000 USD for SO_2_).

Average values for monthly removal of air pollution show a similar pattern across pollutants. January, November, and December were clearly the months where the uptake was lowest for all pollutants (percentages of uptake during the 3 months were 4.58 for CO, 8.45 for NO_2_, 15.15 for PM_10_, 2.69 for O_3_, and 6.75 for SO_2_). Spring and summer (from April to September) were the seasons with higher removal rates in average (percent of uptake during the 2 seasons was 60.96 for CO, 64.25 for NO_2_, 54.43 for PM_10_, 78.90 for O_3_, and 70.46 for SO_2_), although in some cases the highest monthly uptake rate corresponded to other periods (e.g., PM_10_ removal was highest in February, accounting for 10.69 % of total uptake). These patterns in uptake values are normally correlated with the seasonal variation in air pollutants concentrations and the biological cycle of trees (Nowak 1994; Yang et al. 2005). For instance, removal rates of ground-level O_3_ are highest in summer, when concentrations are normally higher due to a more active process of photochemical reaction forming O_3_ as a consequence of warmer temperatures and due to increased leaf surface area and gas exchange at the leaf surface.

### Climate Regulation

The total biophysical value of net carbon sequestration is estimated at 5187 t C year^−1^ (19 036 t CO_2_eq year^−1^) with an economic value of 407 000 USD year^−1^ (Table 2). This total net carbon sequestration is the only value including the effect of GHG emissions of green space maintenance, since disaggregate data by land use was not available. In absolute terms urban green, natural green, and high-density residential are the land use strata contributing the most to total net carbon sequestration (19, 39, and 24 %, respectively). However, considering the ratio net carbon sequestration per land use area, it is the urban green class that shows the highest values among these three land uses (1.24 t ha^−1^ urban green, 0.96 t ha^−1^ natural green, and 0.35 t ha^−1^ high-density residential). Surprisingly, the highest ratio among all land use classes is in the low-density residential stratum (1.33 t ha^−1^).

**Table 2:** Carbon storage and annual carbon sequestration by land use class (urban forests of the municipality of Barcelona, year 2008)

Land use class	Biophysical values						Monetary values	
	Carbon storage		Gross carbon sequestration		Net carbon sequestration		Net carbon sequestration	
	t	SE	t year^−1^	SE	t year^−1^	SE	USD year^−1^	SE
Urban green	26 876	4083	1088	109	1002	100	78 688	7839
Natural green	42 108	4115	2446	207	2099	181	164 804	14 224
Low-density residential	9764	2663	613	169	565	155	44 326	12 173
High-density residential	21 014	2940	1398	157	1282	149	100 630	11 660
Transportation	3876	1213	207	56	196	54	15 366	4250
Institutional	3452	2200	76	43	−64	109	−4995	8518
Commercial/industrial	328	153	32	15	31	14	2409	1086
Intensively used areas	6020	1693	328	65	311	62	24 396	4844
Total	113 437	19 059	6187	819	5422 5187^a^	823	425 625 407 177^a^	64 595

^a^Net carbon sequestration values taking into account GHG emissions of green space maintenance

*SE* standard error

### Air Pollution Due to Biogenic Emissions

The total biophysical value of BVOC emissions is estimated at 183.98 t year^−1^ (Table 3). Similar to the case of carbon sequestration values, results for biogenic emissions show a major contribution of urban green, natural green, and high-density residential land use strata relative to the overall biophysical value for this ecosystem disservice (17.05, 47.46, and 15.32 %, respectively). Urban green, natural green, and low-density residential show to be the strata with the highest relative contribution to BVOC emissions in the city (39, 40, and 35 kg ha^−1^, respectively) considering the ratio BVOC emissions per land use area. Besides, isoprene is clearly the main BVOC emitted (51.8 % of total emissions) in all land use classes (except for institutional), followed by other BVOCs (28.6 %) and monoterpenes (19.6 %).

**Table 3:** Annual BVOC emissions by land use class (urban forests of the municipality of Barcelona, year 2008)

Land use class	Isoprene emissions (t year^−1^)	Monoterpenes emissions (t year^−1^)	Other BVOCs emissions (t year^−1^)	Total BVOC emissions (t year^−1^)
Urban green	16.78	4.94	9.65	31.36
Natural green	38.79	23.65	24.87	87.31
Low-density residential	8.81	1.93	4.06	14.81
High-density residential	17.09	3.20	7.89	28.18
Transportation	4.19	0.57	1.24	6.01
Institutional	0.91	1.18	2.69	4.78
Commercial/industrial	1.13	0.01	0.16	1.29
Intensively used areas	7.66	0.58	2.00	10.24
Total	95.36	36.07	52.56	183.98

### Ecosystem Services Contribution to Air Quality and Climate Change Mitigation

From total biophysical accounts for removal of PM_10_, NO_2_, and CO_2_eq, we estimated the relative contribution of urban forests ecosystem services to air quality and climate change mitigation based on air pollution and GHG emissions levels in the city (Table 4). Our results suggest that the contribution of urban forests to climate change mitigation is very low, accounting for 0.47 % of the overall city-based GHG emissions. If we only account for GHG emissions derived from the sectors that are directly managed by the City Council (reference emissions to meet “Covenant of Mayors” 23 % reduction target and representing 2.10 % of the total emissions) the contribution of urban forest is still modest but yet substantial, accounting for 22.55 % of the emissions. Contributions of urban forests to air quality based only on city emissions differ notably depending on each air pollutant. While the overall contribution of urban forest to NO_2_ removal is low relative to total emissions (0.52 %), its contribution to the removal of PM_10_ amounts to a significant 22.31 %. However, if we account for background pollution levels, the contribution of PM_10_ removal drops to 2.66 % of total PM_10_ pollution levels.

**Table 4:** Contribution of urban forests on air quality and climate change mitigation (year 2008)

Air pollutant	Removal biophysical value (t year^−1^)	Removal monetary value (USD year^−1^)	City-based emissions (t year^−1^)	Background pollution influence (%)	Ecosystem Service contribution (%)	
					City-based emissions	City-based emissions and background pollution
PM_10_	166.01	1 097 964	743.77	88.10	22.32	2.66
NO_2_	54.59	540 745	10 412.94	18.70	0.52	0.43
CO_2_eq	19 036	407 177	4 053 766 84 403^a^	N/A	0.47 22.55^a^	N/A

^a^CO_2_eq emissions from services and activities directly managed by the City Council (“Covenant of Mayors” policy target baseline emissions)

## Discussion

### Urban Forests Potential Contribution to Meet Air Quality Policy Targets

Urban forests effects on air quality are still a subject of intensive research. While positive effects of air purification delivered by vegetation have been estimated at the city scale in many urban areas (e.g., Nowak et al. 2006), pollution concentration can be increased at the site scale (e.g., street canyons) depending upon vegetation configuration, pollutant emissions, or meteorology, showing apparently divergent results on the effectiveness of using urban vegetation for reducing local air pollution hotspots (Pugh et al. 2012; Vos et al. 2013). Likewise, the ability of urban vegetation to remove air pollutants significantly depends on many factors, such as tree health, soil moisture availability, leaf-period, LAI, meteorology, and pollution concentrations.

Our results show that the overall annual air purification rate by urban forests in Barcelona (9.3 g m^−2^ of canopy cover year^−1^) is very similar to US cities like Columbus, Kansas City, or Portland (9.2 g m^−2^ year^−1^), although the PM_10_ removal rate (5.1 g m^−2^ year^−1^) is significantly higher than for these cities (between 3.1 and 3.4 g m^−2^) and closer to cities like Salt Lake City (5.2 g m^−2^), Philadelphia (5.5 g m^−2^), or San Diego (5.6 g m^−2^) (Nowak et al. 2006). The higher removal rates for PM_10_, NO_2_, and O_3_ compared to CO and SO_2_ should be mainly attributable to the almost linear relationship between pollution removal and ambient pollution concentrations considered in the model (pollutant flux equation as *F* = *V*_d_ × *C*). However, very high pollutant concentrations could severely damage vegetation or lead to stomatal closure, reducing air pollution removal ability (Robinson et al. 1998; Escobedo and Nowak 2009). Unfortunately, these environmental thresholds are not yet factored in the i-Tree Eco model.

Our findings also show that the NO_2_ removal rate by urban forests in Barcelona has a meager impact relative to actual city-based emissions (less than 1 %). Therefore, the potential of urban forests to contribute to the compliance of the EU limit is expected to be very low. NO_2_ concentrations in the city derive largely from road transport activity (65.6 % impact according to PECQ 2011). Hence, actions focused on reduction of road traffic, technological change toward less-polluting fuels and the promotion of public transport or cycling utilities are expected to contribute more efficiently to meet policy targets. These actions can also lead to reduction in O_3_ concentrations, as NO_2_ is a precursor chemical to O_3_ formation. PM_10_ removal rate from urban forests is notably higher than NO_2_ rate, whereas city-based emissions of PM_10_ are notably lower, resulting in a substantial impact at the city scale (22.3 % of total city-based emissions). However, the background pollution effect (accounting for 88.1 % of the average annual PM_10_ concentration according to PECQ estimations) drastically reduces the actual impact of the urban forests service (2.7 % of total PM_10_ pollution levels). Yet, we claim that there are still important reasons for which this ecosystem service should be accounted for in local policy decision-making. First, air pollution from particulate matter is a major health problem in Barcelona metropolitan area and recent research suggests that even moderate improvements in air quality are expected to report significant health benefits, together with related economic savings (Pérez et al. 2009). Second, the major role of PM_10_ background pollution in Barcelona air quality might compromise the effectiveness of municipal policies solely based on city emissions abatement. This fact also suggests that measures focused on air quality regulation should be implemented at broader spatial scales, particularly at the metropolitan level. To this end, strong coordination policies between municipal and regional authorities dealing with environmental quality and urban planning are fundamental. Third, the implementation of green infrastructure-based strategies to foster air purification (and other ecosystem services) is a realistic policy option considering the current urban context of Barcelona. I-Tree Eco results show that approximately 3.6 % of the municipality area (364 ha) can be considered as available land for planting. As a complementary alternative, green roofs and walls, yet to be extensively developed in Barcelona, could be particularly appropriate in high-density neighborhoods where ground for planting is extremely scarce. Several studies have quantified the potential of green roofs for air purification in cities at the street canyon (Baik et al. 2012), neighborhood (Currie and Bass 2008), and municipality (Yang et al. 2008) scales, besides their potential to provide many other services and benefits, such as runoff mitigation, noise reduction, or urban cooling (Oberndorfer et al. 2007; Rowe 2011). However, the technical and economic feasibility of green roofs expansion, together with possible trade-offs concerning their maintenance such as water demand, should previously be assessed in Barcelona, especially for existing buildings.

Proper management of existing green space can also contribute to air quality improvement. Yang et al. (2005) lists several factors to consider in strategies for air quality improvement based on green infrastructure, including selection of species (e.g., evergreen versus deciduous trees, dimension, growth rate, leaf characteristics, or air pollution tolerance) and management practices (e.g., intensity of pruning). Previous studies in cities with high levels of air pollution (e.g., Nowak et al. 2006; Escobedo and Nowak 2009) suggest that meteorological conditions, mixing-layer height (the atmospheric layer which determines the volume available for the dispersion of pollutants, see Seibert et al. 2000 for a complete definition), and vegetation characteristics (e.g., proportion of evergreen leaf area, in-leaf season, and LAI) are important factors defining urban forest effects on air quality. Further research is needed to advance our understanding of the role of morphology, function, and ecophysiology of vegetation in air purification (Manning 2008).

A further critical issue concerns the understanding of trade-offs with other ecosystem services or disservices. For example, urban parks are considered very relevant ecosystems for the provision of outdoor recreation and other cultural services in cities (Chiesura 2004). However, highly maintained parks might remove less air pollutants and CO_2_ (due to emissions from maintenance activities, Nowak et al. 2002b) than natural areas that are not intensively managed, but which can be perceived as unpleasant or even dangerous, hence providing few cultural services (Lyytimäki and Sipilä 2009; Escobedo et al. 2011). Likewise, urban tree species with high potential for air purification can be highly invasive as well in certain cities (Escobedo et al. 2010). More generally, many specific environmental factors (e.g., soil condition, climate, water availability, or longevity of the species) should be considered in urban forest management to avoid conflicts with other municipal sustainability goals (Yang et al. 2005; Escobedo et al. 2011).

The i-Tree Eco model could not provide reliable results on O_3_ and CO formation rates associated to the quantified BVOC emissions. However, as mentioned above, CO levels in Barcelona (2.7 mg m^3^ for a daily 8-h average was the highest measure in 2011 according to ASPB air quality report 2011) have been historically far below the EU reference value (10 mg m^3^ daily 8-h average). Thus, it is unlikely that urban forests may compromise in any significant form the compliance of air quality relative to CO target. In contrast, ground-level O_3_ levels have surpassed the EU reference value (120 μg m^−3^ daily 8-h average) at some monitoring stations in the last decade, even if the allowed exceedences have never been reached. Although O_3_ concentrations have remained steady in the last decade within the municipality of Barcelona, O_3_ formation due to BVOC emissions might cause air quality problems in the long term, where BVOC emissions are expected to increase due to global warming (Peñuelas and Llusià 2003). Nevertheless, several studies point out that the selection of low BVOC-emitting tree species can contribute positively in O_3_ concentrations in urban areas because BVOC emissions are temperature dependent and trees generally lower air temperatures (Taha 1996; Nowak et al. 2000; Paoletti 2009). Chaparro and Terradas (2009) identified some of the tree and shrub species in Barcelona emitting less BVOC per leaf biomass. These include genera such as *Pyrus*, *Prunus*, *Ulmus*, and *Celtis*.

### Urban Forests Potential Contribution to Meet Climate Change Mitigation Policy Targets

Some authors suggest that global climate regulation does not stand amongst the most relevant ecosystem services in the urban context because cities can benefit from carbon offsets performed by ecosystems located elsewhere (Bolund and Hunhammar 1999). However, other authors argue that urban forests can play an important role in mitigating the impacts of climate change if compared to other policies at the city level (McHale et al. 2007; Escobedo et al. 2010; Zhao et al. 2010; Liu and Li 2012).

The estimated net annual carbon sequestration per hectare of Barcelona (536 kg ha^−1^ year^−1^) is very similar to cities such as Baltimore (520 kg ha^−1^ year^−1^) or Syracuse (540 kg ha^−1^ year^−1^) (Nowak and Crane 2002). It should be noted that an analysis of the overall contribution of urban green infrastructure to climate change mitigation should also account for the effects of vegetation on micro-climate regulation, which can indirectly avoid CO_2_ emissions through energy saving in buildings for heating and cooling (Nowak and Crane 2002). Hence, our quantification likely underestimates the total contribution of urban forests to climate change mitigation. Analyzing the results by land use, urban green and natural green strata are relevant for the supply of climate regulation service due to the high vegetative cover compared to the other land use classes. High-density residential stratum also showed an important rate in net carbon sequestration, mainly attributable to its large total area (36 % of the municipality) and probably, to a lesser extent, to the high presence of street trees in these neighborhoods. Finally, the high ratio of net carbon sequestration per area observed in the low-density residential stratum could be attributed to the high presence of private gardens in these areas, together with low decomposition emissions due to healthier vegetation.

In line with the results obtained in other urban studies (Pataki et al. 2009; Liu and Li 2012), our findings show that direct net carbon sequestration in Barcelona makes a very modest contribution to climate change mitigation relative to total city-based annual GHG emissions (0.47 %). Nevertheless, if we only account for the GHG emissions from services and activities directly management by the City Council (baseline emissions for the 23 % reduction target from the “Covenant of Mayors”), the contribution of urban forest is notably higher (22.55 %). Similar green infrastructure-based strategies as specified for air quality improvement could also improve the contribution of urban forests to offset GHG emissions and meet the urban policy target of 23 % reduction until 2020.

### Limitations and Caveats

The main advantages of the i-Tree Eco model stem from the reliance on locally measured field data and standardized peer-reviewed procedures to measure urban forest regulating ecosystem services in cities (Nowak et al. 2008a). Favored by its status as an open access model, it has been widely applied across the world (e.g., Nowak and Crane 2002; Yang et al. 2005; Nowak et al. 2006; Currie and Bass 2008; Escobedo and Nowak 2009; Dobbs et al. 2011; Liu and Li 2012).

However, i-Tree Eco has some limitations that should be taken into account when analyzing its outcomes. First, the model is especially designed for US case studies and its application in other countries is subject to some restrictions, as stated in the user’s manual. For instance, although the i-Tree Eco database has over 5000 species, it did not include some tree and shrub species sampled in Barcelona, which then needed to be added to the database. Likewise, monetary valuations of air purification and climate regulation services are based on the literature (see “Materials and Methods” section) which mainly apply to the US context and, hence, should be considered a rough estimation for Barcelona. However, these values are direct multiplier to the biophysical accounts, thus they can be easily adjusted to the case study context when data will be available. Another important limitation applying to i-Tree Eco and most dry deposition models is the level of uncertainty involved in the quantification of the air pollution removal rates due to the complexity of this process (Pataki et al. 2011). For instance, some sources of uncertainty include non-homogeneity in spatial distribution of air pollutants, particle re-suspension rates, transpiration rates, or soil moisture status (Manning 2008). Though the model outputs match well with field measured deposition velocities for urban forests, the model analyzes average effects across a city, not local variations in removal caused by local meteorological and pollution differences. However, these local fine-scale input data are often missing from urban areas and empirical data on the actual uptake of pollutants by urban vegetation are still limited (Pataki et al. 2011; Setälä et al. 2013), which makes a more accurate modeling of this ecosystem service unfeasible at the moment. For a sensitivity analysis of the i-Tree Eco deposition model see Hirabayashi et al. (2011). Estimation errors in climate regulation service values include the uncertainty from using biomass equations and conversion factors as well as measurement errors (Nowak et al. 2008a). For example, there are limited biomass equations for tropical tree species (e.g., palm trees), some of them present in Barcelona. Estimates of carbon sequestration and storage also include uncertainties from factors such as urban forests maintenance (e.g., intensity of pruning), tree decay, or restricted rooting volumes, which are not accounted for in the model’s estimations (Nowak et al. 2008a; Pataki et al. 2011). BVOC emissions are estimated based on species factors and meteorological conditions (i.e., air temperature and daylight) but the uncertainty of the estimate is unknown. As mentioned in previous sections, O_3_ and CO formation rates from BVOC emissions cannot be estimated with an acceptable level of reliability.

Therefore, the results presented in this paper should be considered as an approximate estimation rather than a precise quantification of the ecosystem services and disservices delivered by the urban forests of Barcelona. However, these estimates allow one to evaluate the contribution of urban forests in air quality and climate change mitigation in the city, and also to derive implications and recommendations for urban decision-making.

## Conclusion

Regulating ecosystem services provided by urban forests have been widely analyzed in many cities across the world. However, the potential effectiveness of urban forests in air quality improvement and climate change mitigation is still object of debate, mainly due to the multiple factors and uncertainties involved in the actual delivery of these ecosystem services in cities, especially at the patch or site scale. Further, this potential is barely reflected in terms of its contribution to meet specific policy targets.

Our findings show that the contribution of urban forests regulating services to abate pollution is substantial in absolute terms (305.6 t of removed air pollutants year^−1^ and 19 036 t CO_2_eq year^−1^), yet modest when compared to overall city levels of air pollution and GHG emissions (2.66 % for PM_10_, 0.43 % for NO_2_, and 0.47 % for CO_2_eq). Our research further shows that the effectiveness of green infrastructure-based strategies to meet environmental policy targets can vary greatly across pollutants. For example, our results suggest that NO_2_ removal potential is unlikely to contribute in any substantial way to the compliance of current EU reference values. Therefore, for combating air pollution of NO_2_, synergies between green infrastructure strategies and NO_2_ emission curbing strategies (e.g., targeting road traffic) need to be searched and implemented in order to effectively deal with air quality regulations. On the other hand, PM_10_ removal potential should not be neglected in urban policy-making. Its contribution to the compliance with the current EU reference value can be substantial and potentially more effective than other local policies based on emissions abatement due to the importance of background pollution in Barcelona’s PM_10_ levels.

Net carbon sequestration by urban forests has a very low influence when compared to total annual GHG city emissions, but our results suggest that it can contribute considerably to meet the 23 % GHG emissions reduction policy target until 2020, which only applies for emissions derived from services and activities directly managed by the City Council (2.10 % of total emissions).

We determine that the implementation of green infrastructure-based strategies at the municipal level (as is aimed by the *Barcelona Green Infrastructure and Biodiversity Plan 2020*) would have a limited effect on local air quality levels and GHG emissions offsets, yet they would play a non-negligible complementary role to other policies intended to meet air quality (especially for PM_10_ levels) and climate change mitigation policy targets in Barcelona, fostering as well the provision of other important urban ecosystem services (e.g., urban temperature regulation, stormwater runoff mitigation, and recreational opportunities) at no additional monetary costs. We conclude that, in order to be effective, green infrastructure-based strategies to abate pollution in cities should be implemented at broader spatial scales (i.e., metropolitan area). However, it is critical that policy-makers consider an integrated approach in green infrastructure management, where possible trade-offs with other ecosystems services, disservices, and urban sustainability goals are fully acknowledged.

## Footnotes

1 Here “green space” corresponds to those areas with vegetation (e.g., urban parks, gardens, and other green areas) directly managed by the City Council. It includes also the natural and semi-natural areas of the Collserola Park, but it excludes green elements such as single street trees or private gardens.

2
^“^Green infrastructure is a concept addressing the connectivity of ecosystems, their protection and the provision of ecosystem services, while also addressing mitigation and adaptation to climate change” (EEA 2011).
